# The Association between Standardized Serum 25-Hydroxyvitamin *D* Concentration and Risk of Anemia: A Population-Based Cross-Sectional Study

**DOI:** 10.1155/2022/8384306

**Published:** 2022-10-13

**Authors:** Yang Zhang, Xiaotong Wang, Jia Wang, Weiwei Hu, Xiaolu Song, Ding Yuan, Xianliang Yan

**Affiliations:** ^1^Department of Critical Care Medicine, The Affiliated Hospital of Xuzhou Medical University, Xuzhou 221006, Jiangsu, China; ^2^Department of Critical Care Medicine, Suining County People's Hospital, Xuzhou 221200, Jiangsu, China; ^3^Department of Cardiology, The Affiliated Hospital of Xuzhou Medical University, Xuzhou 221006, Jiangsu, China; ^4^Department of Nephrology, The Affiliated Hospital of Xuzhou Medical University, Xuzhou 221006, Jiangsu, China; ^5^Department of Emergency Medicine, The Affiliated Hospital of Xuzhou Medical University, Xuzhou 221006, Jiangsu, China; ^6^Department of Emergency Medicine, Suining County People's Hospital, Xuzhou 221200, Jiangsu, China

## Abstract

The relationship between standardized serum 25-hydroxyvitamin *D* (25(OH)D) concentration and incident anemia in the United States (U.S.) is unclear. The purpose of our study was to examine the association between serum 25(OH)D and anemia risk. We performed a cross-sectional analysis of the U.S. population participating in the National Health and Nutrition Examination Survey (NHANES) between 2001 and 2018. A generalized linear model and restricted cubic spline (RCS) plot curve were constructed to assess the relationship between serum 25(OH)D concentration and anemia incidence. Additionally, the association between serum 25(OH)D concentration and red blood cell (RBC) count and hemoglobin (HB) levels was investigated using generalized additive models with smooth functions. Subgroup analysis also was performed. A total of 29933 individuals were included in our research. After adjusting for known confounding variables, compared with the lowest quartile, the odds ratios (ORs) with 95% confidence intervals (CIs) for association of serum 25(OH)D with anemia across the second, third, and fourth quartiles were 0.735 (0.651, 0.829), 0.527 (0.461, 0.602), and 0.696 (0.611, 0.792), respectively. Serum 25(OH)D concentration was associated with anemia risk in a U-shaped pattern, as shown by an RCS plot (*P* for nonlinearity <0.001). In addition, RBC count and Hb levels initially increased and then decreased as serum 25(OH)D levels increased. Serum 25(OH)D concentration and risk of anemia are associated with a U-shaped curve in the U.S. general population. Serum 25(OH)D concentration in the range 59.7–70.3 nmol/l was associated with anemia incidence <1. Therefore, the risk of anemia can be reduced by close monitoring and appropriate vitamin *D* supplementation.

## 1. Introduction

Worldwide, anemia affects one out of every three people and is estimated to represent a greater global disease burden than major depression and chronic respiratory illnesses combined [1, 2]. Symptoms of anemia include fatigue, weakness, cognitive decline, and even death, and the condition is more common in women [3]. Anemia pathogenesis involves an imbalance between red blood cell loss and production, which may be due to ineffective or inadequate erythropoiesis and/or excessive red blood cell loss [1]. The most common conditions causing anemia are iron deficiency, thalassemia and hemoglobinopathies, folate deficiency, and parasitic diseases [4].

As a fat-soluble vitamin, vitamin *D* is naturally found in very few foods; the canonical role of vitamin *D* is to regulate the balance of calcium and phosphorus metabolism and minerals in bones [5]. Vitamin *D* deficiency has been linked to anemia [6–9], since vitamin *D* is found in the bone marrow and can promote red blood cell production [10–12]. The main ring form of vitamin *D*, 25(OH)D is created by hydroxylation of vitamin *D*, which can be taken in from exogenous sources or synthesized in the liver [13]. In recent years, the relationship between 25(OH)D and anemia has attracted increasing attention. Many previous studies have shown a correlation between 25(OH)D and anemia in chronic kidney disease as well as sickle cell anemia [14–18]; however, few recent studies have explored the link between serum 25(OH)D levels and anemia risk, and previous studies had small sample sizes or concomitant disease interference.

Due to the detrimental effects of anemia, recognizing risk factors for anemia development and devising measures to promptly avoid or control its negative consequences would be highly advantageous. According to epidemiological research, the relationship between serum 25(OH)D and anemia risk in the United States (U.S.) population remains unclear. Therefore, in this study, we analyzed data from the Nutrition and Health Examination Survey (NHANES) 2001–2018 to investigate the link between serum 25(OH)D and anemia incidence in the U.S. general population.

## 2. Materials and Methods

### 2.1. Study Population

NHANES is an American cross-sectional survey that collects data on the health and nutrition of the general population through stratified multistage random sampling (https://www.cdc.gov/nchs/nhanes/). Eight two-year cycles (2001–2018) of data from NHANES were used for this analysis. Participants with insufficient serum 25(OH)D or anemia data were excluded (*n* = 10632 and 146, respectively). Additionally, participants <18 years old and with missing demographic or biochemical data were excluded (*n* = 41606). Finally, this research included a total of 29933 individuals. Data on parathyroid hormone (PTH) levels, history of osteoporosis, and arthritis or rheumatism obtained by the affiliated hospital of Xuzhou Medical University between 2012 and 2022 were also included and analyzed in this study. NHANES was authorized by the National Center for Health Statistics study ethical review board, and each participant provided signed written informed permission [19]. All tests were taken at a mobile testing facility on-site.

### 2.2. Serum 25(OH)D Concentrations

Blood samples were collected during the examination, centrifuged, divided, and frozen at –70°C on-site, then shipped on dry ice to a central laboratory, where they were stored at –70°C until analysis. After acetonitrile-based extraction, a radioimmunoassay kit (DiaSorin, Stillwater, MN) was used to measure serum 25(OH)D levels at the National Center for Environmental Health (Atlanta, GA); however, due to concern about bias and imprecision of the DiaSorin radioimmunoassay (RIA), the Centers for Disease Control (CDC) developed regression equations to convert RIA values to liquid chromatography with tandem mass spectrometry (LC-MS/MS) equivalents for NHANES 2001–2006. In addition, the CDC laboratory analyzed serum 25(OH)D metabolites from 2007 to 2018, using LC-MS/MS, and calculated total serum 25(OH)D (nmol/L) as the sum of 25(OH)D3 and 25(OH)D2, excluding the C3-epi-25(OH)D3 metabolite. Detailed information and procedures are described on the NHANES website (https://wwwn.cdc.gov/nchs/nhanes/vitamind/analyticalnote.aspx).

### 2.3. Covariates

The following covariates were included in the study: age, sex, race/ethnicity, family poverty income ratio (PIR), education level, marital status, hypertension, diabetes mellitus (DM), smoking status, and alcohol consumption status; history of coronary heart disease (CHD), congestive heart failure (CHF), angina pectoris, heart attack, stroke, chronic kidney diseases (CKD), and osteoporosis; body mass index (BMI); waist circumference; folic acid, vitamin B12, vitamin C, iron; and serum iron, calcium, phosphorus, and PTH; and number of days with arthritis or rheumatism. In addition, red blood cell (RBC) numbers and hemoglobin (Hb) levels of participants were collected. During the home interview, the following data were self-reported by participants: age, sex, ethnicity, education level, marital status, smoking status, alcohol consumption status, and dietary intake; history of CHD, CHF, angina pectoris, heart attack, stroke, CKD, and osteoporosis; and number of days with arthritis or rheumatism. Data on serum iron, calcium, phosphorus, and PTH were obtained from laboratory tests. More information about the variables analyzed in this research can be found at https://www.cdc.gov/nchs/nhanes/.

### 2.4. Anemia Ascertainment

The World Health Organization defines anemia as hemoglobin level <13 g/dL in men and <12 g/dL in women [20]. A Beckman Coulter DxH 800 instrument in the NHANES mobile examination center produces a complete blood count from blood specimens, which provided blood cell distributions for all participants. Refer to the Laboratory Method Files section for a detailed description of the laboratory methods used.

### 2.5. Statistical Analysis

All analyses were performed using *R* version 3.6.4 (R Foundation for Statistical Computing, Vienna, Austria) and Stata version 13.0 (Stata Corporation, College Station, TX, USA). Samples with missing covariate data were excluded from this study. *P*-value <0.05 was regarded as statistically significant. Serum 25(OH)D levels were divided into quartiles, and the lowest quartile served as the reference group (Q1). All estimates were calculated accounting for NHANES sample weights. Continuous variables are expressed as mean (standard deviation) and categorical variables are presented as numbers (%). Weighted linear regression models (continuous variables) and weighted chi-square tests (categorical variables) were used to assess the significance of differences. Multivariate logistic regression analysis was used to investigate the relationship between serum 25(OH)D levels and anemia risk. Model 1 was adjusted for age and sex. Model 2 was adjusted for model 1 variables plus race/ethnicity, education level, marital status, family PIR, smoking status, alcohol consumption status, history of hypertension, and DM. Finally, model 3 was adjusted for model 2 variables plus history of CHD, CHF, angina pectoris, heart attack, stroke, and CKD; BMI; waist circumference; folic acid, vitamin B12, vitamin C, iron; and serum iron, calcium, and phosphorus, as our core model.

## 3. Results

### 3.1. Baseline Characteristics

Table 1 shows the baseline characteristics of the research participants. The incidence of anemia in this group was 8.3%. The characteristics of the participants were subclassified based on serum 25(OH)D quartiles (Q1: 6.31–44.4 nmol/L; Q2: 44.5–60.6 nmol/L; Q3: 60.7–77.7 nmol/L; and Q4: 77.8–422 nmol/L). There were significant differences in age, sex, race, family PIR, education level, marital status, smoking status, alcohol consumption status, hypertension, DM, CHF, stroke, CKD, BMI, waist circumference, RBC, Hb, serum iron, calcium, phosphorus, mean energy intake, protein intake, folic acid intake, vitamin B12 intake, and iron intake among the Q1, Q2, Q3, and Q4 groups. Compared with Q1, Q3, and Q4 groups, the Q2 group had a lower proportion of participants with hypertension, heart attack, stroke, and CKD, as well as the highest RBC counts; however, compared with the Q1, Q2, and Q4 groups, a lower proportion of individuals in the Q3 group had DM, CHF, angina, and anemia, while they had the highest levels of Hb, mean energy intake, protein intake, folic acid intake, vitamin B12 intake, and iron intake. In addition, compared with Q1, Q2, and Q3, participants in Q4 were older and had the highest levels of family PIR, calcium, phosphorus, serum iron, and vitamin C intake, and had lower BMI and waist circumference. The basic characteristics of the 959 participants from the affiliated hospital of Xuzhou Medical University are shown in Supplementary Table 1, including the levels of PTH, history of osteoporosis, and number of days with arthritis or rheumatism.

### 3.2. Association between Serum 25(OH)D and Anemia

**Figure 1:**
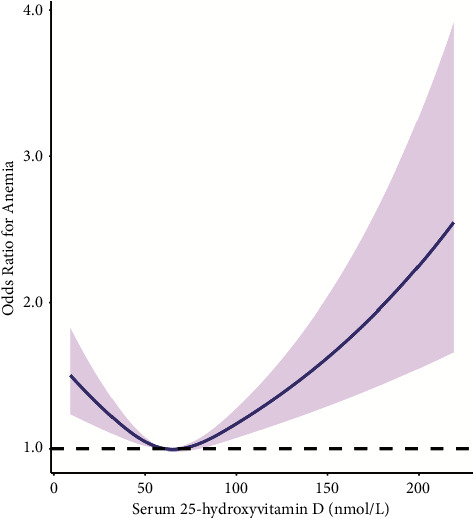
Restricted cubic spline plot of the association between serum 25(OH)D and the incidence of anemia.

The findings of multivariate logistic regression analysis of the relationship between serum 25(OH)D and anemia are presented in Table 2. After adjusting for interfering factors, the odds ratios (ORs) with 95 percent confidence intervals (CIs) for association of serum 25(OH)D with anemia across quartiles two, three, and four were 0.735 (0.651, 0.829), 0.527 (0.461, 0.602), and 0.696 (0.611, 0.792), compared with Q1. As shown by the restricted cubic spline (RCS) plot, serum 25(OH)D was associated with anemia prevalence with a U-shaped curve (*P* for nonlinearity <0.001, Figure 1). As serum 25(OH)D concentrations increased, the risk of anemia initially decreased significantly; risk of anemia was lowest when serum 25(OH)D concentrations reached 65.0 nmol/*L*, then the curve showed an upward trend. In the fully adjusted model including PTH, history of osteoporosis, and number of days with arthritis or rheumatism, the ORs and 95 percent CIs for anemia incidence across the serum 25(OH)D quartiles were 0.771 (0.449, 1.326), 0.499 (0.267, 0.932), and 0.596 (0.323, 1.097), compared with the first quartile of serum 25(OH)D (Supplementary Table 2). Additionally, RCS plots constructed after adjusting for PTH, history of osteoporosis, and number of days with arthritis or rheumatism as covariates also displayed U-shaped curves (Supplementary Figure 1).

### 3.3. Associations of Serum 25(OH)D with RBC Count and Hb Level

**Figure 2:**
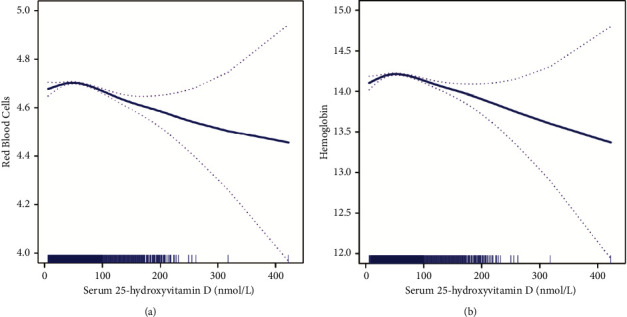
Associations of serum 25(OH)D with hemoglobin levels and red blood cell counts. (a) Association between serum 25(OH)D and hemoglobin level. (b) Association between serum 25(OH)D and red blood cell count.

Generalized additive models with smooth functions suggested nonlinear relationships between serum 25(OH)D and risk of anemia. As serum 25(OH)D levels increased, both RBC count and Hb level first increased and then decreased (Figures 2(a) and 2(b)). Additionally, generalized additive models with smooth functions generated following adjustment for PTH, history of osteoporosis, and number of days with arthritis or rheumatism as covariates also showed an increase, after declining (Supplementary Figures 2(a) and 2(b)).

### 3.4. Subgroup Analyses

Subgroup analyses, stratified by age, sex, race/ethnicity, hypertension, DM, and obesity were conducted to estimate the associations between serum 25(OH)D and incident anemia. The results demonstrated U-shaped relationships between serum 25(OH)D and risk of anemia in individuals of all ages, male or female, who were Mexican American, Other Hispanic, Non-Hispanic Black, or Non-Hispanic White, without hypertension, without DM, and never participated, with or without hypertension, with or without diabetes, and whether or not obese (Table 3). There were significant interactions of age, sex, race/ethnicity, hypertension, DM, and obesity with the associations with serum 25(OH)D in subgroup analyses. In addition, we performed subgroup analysis stratified by age, sex, hypertension, DM, and obesity, to examine the correlation between serum 25(OH)D and the risk of anemia, after adjusting for variables, including PTH, history of osteoporosis, and number of days with arthritis or rheumatism (Supplementary Table 3).

## 4. Discussion

In recent years, studies of the relationship between 25(OH)D and anemia have focused on sickle cell anemia, and have demonstrated that 25(OH)D supplementation is helpful in treatment of sickle cell anemia [16, 18, 21–23]; however, the overall relationship between 25(OH)D and anemia, particularly its trend, has not been clarified. Our study revealed a U-shaped relationship between serum 25(OH)D levels and risk of anemia, and with increased 25(OH)D level, hemoglobin level, and RBC counts the association showed a downward trend, suggesting that caution should be applied in the use of 25(OH)D supplementation to treat anemia. Insufficient 25(OH)D levels can reduce local calcitriol production in the bone marrow and limit erythropoiesis. As calcitriol has a synergistic effect with endogenous erythropoietin, it can up-regulate the expression of erythropoietin receptors on erythrocyte progenitor cells [24–26]; however, there is no clear explanation for the relationship between higher levels of 25(OH)D and increased risk of anemia, which appears contradictory to the established effect of 25(OH)D. We consider that this anomalous outcome may be attributable to concomitant diseases in participants, many of which led to end-stage metabolic disorders at physiological hormonal and cellular levels. It is likely that 25(OH)D inhibits other hematopoietic processes through a feedback system, thereby causing a decrease in red blood cell counts and hemoglobin levels, as well as an increase in the risk of anemia. According to our study, when serum 25(OH)D level is controlled between 59.7 and 70.3 nmol/l, the risk of anemia can be effectively reduced; this finding requires further confirmation in basic experiments.

Anemia is considered an independent cause of morbidity and mortality in the elderly, and its incidence varies with sex, race, and age [27, 28]. Anemia can reduce the ability of adults to work and affect the growth and development of children and adolescents, and is more common in women [29, 30]. Similarly, 25(OH)D may have different basal levels and sensitivities in different populations. For example, Atkinson et al. showed that black children have a lower 25(OH)D threshold than white children [8]. Our subgroup analysis also suggested significant interactions of anemia with age, sex, race/ethnicity, hypertension, diabetes, obesity, and serum 25(OH)D. For the Hispanics (other) ethnic category, the 25(OH)D threshold was significantly lower than that for other races.

Immune diseases are a cause of anemia, and are often accompanied by abnormal metabolism of vitamin *D* and PTH, leading to osteoporosis [31, 32]. Further, low levels of 25(OH)D may increase the risk of immune-related diseases because they exert an immunomodulatory effect through nuclear vitamin *D* receptor expressed by antigen-presenting cells and activated *T* and B cells [33, 34]. Therefore, we screened the database for data from patients with immune diseases and hyperparathyroidism; after adjusting for these covariates, the results were generally consistent with those above, confirming that arthritis and rheumatism are not additional risk factors for reduced plasma 25(OH)D concentrations [35]. Notably, 25(OH)D levels in patients with arthritis and rheumatism were higher than those in the whole group when anemia risk was in the trough, which may be due to the effects of the pathological processes underlying arthritis or rheumatism on anemia, or the regulation of 25(OH)D by PTH; however, neither significantly affected the overall trend relationship between anemia and 25(OH)D. Therefore, the use of vitamin *D* supplementation to treat anemia needs to be personalized for different populations, particularly in patients with comorbidities that may affect vitamin *D* metabolism. In summary, our study shows that there is no simple linear relationship between 25(OH)D and risk of anemia, and that age, sex, race, hypertension, diabetes, arthritis, rheumatism, and obesity all interact with 25(OH)D levels. Further basic and prospective experiments are needed to further explore the relationship between 25(OH)D and the mechanism underlying anemia development.

Our study has some limitations. First, we used the NHANES public database for this analysis, which covers the years 2001 to 2018. To verify our findings, participants from other nations should be recruited. Second, a retrospective study has the disadvantage of introducing bias to some relevant results. Third, a study of the mechanisms underlying serum 25(OH)D levels and anemia incidence is also necessary.

## 5. Conclusion

In conclusion, the relationship between serum 25(OH)D and risk of anemia presents as a U-shaped curve. An inflection point for serum 25(OH)D was observed in our study; the incidence of anemia was lowest when the serum 25(OH)D level was 65.0 nmol/l, and anemia risk was <1 when serum 25(OH)D levels were between 59.7 and 70.3 nmol/l. Therefore, with close monitoring and adequate vitamin *D* supplementation, the risk of anemia can be reduced.

## Figures and Tables

**Table 1:** Study population data according to serum 25(OH)D quartiles.

Serum 25(OH)D	Total	Q1	Q2	Q3	Q4	*P*-value
Age, years	47.319 ± 0.218	44.127 ± 0.288	45.035 ± 0.323	47.076 ± 0.279	51.095 ± 0.294	<0.001

Gender, %						
Male	14830 (49.5%)	3479 (11.6%)	4020 (13.4%)	4032 (13.5%)	3299 (11.0%)	<0.001
Female	15103 (50.5%)	4035 (13.5%)	3461 (11.6%)	3432 (11.5%)	4175 (13.9%)	

Race, %						
Mexican American	4818 (16.1%)	1505 (5.0%)	1630 (5.4%)	1141 (3.8%)	542 (1.8%)	<0.001
Other Hispanic	2306 (7.7%)	523 (1.7%)	718 (2.4%)	639 (2.1%)	426 (1.4%)	
Non-Hispanic Black	5912 (19.8%)	3116 (10.4%)	1338 (4.5%)	790 (2.6%)	668 (2.2%)	
Non-Hispanic White	14445 (48.3%)	1673 (5.6%)	3158 (10.6%)	4333 (14.5%)	5281 (17.6%)	
Other race	2452 (8.2%)	697 (2.3%)	637 (2.1%)	561 (1.9%)	557 (1.9%)	

Family PIR	3.108 ± 0.030	2.582 ± 0.036	2.957 ± 0.039	3.228 ± 0.035	3.422 ± 0.038	<0.001

Education level, %						
Less than high school	6876 (23.0%)	2019 (6.7%)	1944 (26.0%)	1641 (5.5%)	1272 (4.2%)	<0.001
High school	2835 (9.5%)	717 (2.4%)	677 (2.3%)	646 (2.2%)	795 (2.7%)	
More than high school	20222 (67.6%)	4778 (16.0%)	4860 (16.2%)	5177 (17.3%)	5407 (18.1%)	

Marital status, %						
Having a partner	18490 (61.8%)	4016 (13.4%)	4674 (15.6%)	4947 (16.5%)	4853 (16.2%)	<0.001
No partner	6415 (21.4%)	1680 (5.6%)	1505 (5.0%)	1497 (5.0%)	1733 (5.8%)	
Unmarried	5028 (16.8%)	1818 (6.1%)	1302 (4.3%)	1020 (3.4%)	888 (3.0%)	

Hypertension, %						
No	17111 (57.2%)	4243 (14.2%)	4515 (15.1%)	4383 (14.6%)	3970 (13.3%)	<0.001
Yes	12822 (42.8%)	3271 (10.9%)	2966 (9.9%)	3081 (10.3%)	3504 (11.7%)	

DM, %						
No	24826 (82.9%)	6059 (20.2%)	6202 (20.7%)	6308 (21.1%)	6257 (20.9%)	<0.001
Yes	5107 (17.1%)	1455 (4.9%)	1279 (4.3%)	1156 (3.9%)	1217 (4.1%)	

Smoker, %						
No	16148 (53.9%)	4183 (14.0%)	4168 (13.9%)	3905 (13.0%)	3892 (13.0%)	<0.001
Former	7669 (25.6%)	1462 (4.9%)	1806 (6.0%)	2139 (7.1%)	2262 (7.6%)	
Now	6116 (20.4%)	1869 (6.2%)	1507 (5.0%)	1420 (4.7%)	1320 (4.4%)	

Alcohol user, %						
Never	3981 (13.3%)	1228 (4.1%)	1030 (3.4%)	851 (2.8%)	872 (2.9%)	<0.001
Former	5146 (17.2%)	1307 (4.4%)	1361 (4.5%)	1292 (4.3%)	1186 (4.0%)	
Mild	10340 (34.5%)	2210 (7.4%)	2491 (8.3%)	2705 (9.0%)	2934 (9.8%)	
Moderate	4636 (15.5%)	1178 (3.9%)	1070 (3.6%)	1204 (4.0%)	1184 (4.0%)	
Heavy	5830 (19.5%)	1591 (5.3%)	1529 (5.1%)	1412 (4.7%)	1298 (4.3%)	

CHD, %						
No	28680 (95.8%)	7258 (24.2%)	7175 (24.0%)	7161 (23.9%)	7086 (23.7%)	0.095
Yes	1253 (4.2%)	256 (0.9%)	306 (1.0%)	303 (1.0%)	388(1.3%)	

CHF, %						
No	29054 (97.1%)	7263 (24.3%)	7278 (24.3%)	7283 (24.3%)	7230 (24.2%)	<0.001
Yes	879 (2.9%)	251 (0.8%)	203 (0.7%)	181 (0.6%)	244 (0.8%)	

Angina, %						
No	29089 (97.2%)	7295 (24.4%)	7267 (24.3%)	7276 (24.3%)	7251 (24.2%)	0.166
Yes	844 (2.8%)	219 (0.7%)	214 (0.7%)	188 (0.6%)	223 (0.7%)	

Heart attack, %						
No	28665 (95.8%)	7209 (24.1%)	7187 (24.0%)	7138 (23.8%)	7131 (23.8%)	0.239
Yes	1268 (4.2%)	305 (1.0%)	294 (1.0%)	326 (1.1%)	343 (1.1%)	

Stroke, %						
No	28877 (96.5%)	7253 (24.2%)	7256 (24.2%)	7215 (24.1%)	7153 (23.9%)	0.001
Yes	1056 (3.5%)	261 (0.9%)	225 (0.8%)	249 (0.8%)	321 (1.1%)	

CKD, %						
No	24689 (82.5%)	6161 (20.6%)	6321 (21.1%)	6287 (21.0%)	5920 (19.8%)	<0.001
Yes	5244 (17.5%)	1353 (4.5%)	1160 (3.9%)	1177 (3.9%)	1554 (5.2%)	

BMI, kg/m^2^	28.844 ± 0.072	30.960 ± 0.137	29.571 ± 0.132	28.492 ± 0.105	27.378 ± 0.091	<0.001

Waist circumference, cm	98.842 ± 0.181	102.572 ± 0.335	100.492 ± 0.319	98.404 ± 0.254	95.823 ± 0.265	<0.001

RBC, million cells/ul	4.714 ± 0.007	4.724 ± 0.010	4.762 ± 0.009	4.750 ± 0.009	4.642 ± 0.010	<0.0001

Hb, g/dl	14.350 ± 0.023	14.093 ± 0.033	14.438 ± 0.028	14.504 ± 0.027	14.300 ± 0.034	<0.0001

Serum iron, ug/dl	87.784 ± 0.348	80.342 ± 0.549	87.907 ± 0.594	88.667 ± 0.498	91.244 ± 0.591	<0.001

Calcium, mg/dl	9.435 ± 0.006	9.390 ± 0.009	9.422 ± 0.008	9.439 ± 0.007	9.469 ± 0.009	<0.001

Phosphorus, mg/dl	1.208 ± 0.002	1.204 ± 0.003	1.204 ± 0.003	1.208 ± 0.003	1.216 ± 0.003	0.005

Mean energy intake, kcal	2137.327 ± 7.357	2071.327 ± 13.574	2150.599 ± 13.724	2204.818 ± 12.138	2106.481 ± 12.942	<0.001

Protein intake, g	83.081 ± 0.323	78.920 ± 0.512	83.956 ± 0.541	85.968 ± 0.571	82.309 ± 0.552	<0.001

Folic acid intake, mcg	189.390 ± 1.525	168.985 ± 2.166	191.162 ± 2.509	200.219 ± 2.819	190.416 ± 2.712	<0.001

Vitamin B12 intake, mcg	5.310 ± 0.050	4.629 ± 0.095	5.275 ± 0.074	5.686 ± 0.091	5.403 ± 0.072	<0.001

Vitamin C intake, mg	83.903 ± 0.872	82.746 ± 1.461	82.011 ± 1.264	84.748 ± 1.207	85.248 ± 1.470	0.189

Iron intake, mg	15.361 ± 0.072	14.070 ± 0.110	15.322 ± 0.111	16.057 ± 0.129	15.528 ± 0.123	<0.001

Anemia, %						
No	27438 (91.7%)	6583 (22.0%)	6898 (23.0%)	7043 (23.5%)	6914 (23.1%)	<0.001
Yes	2495 (8.3%)	931 (3.1%)	583 (1.9%)	421 (1.4%)	560 (1.9%)	

Q1, 6.31–44.4 nmol/L; Q2, 44.5–60.6 nmol/L; Q3, 60.7–77.7 nmol/L; Q4, 77.8–422 nmol/L; Serum 25(OH)D, serum 25-hydroxyvitamin *D*; Family PIR, family poverty income ratio; DM, diabetes mellitus; CHD, coronary heart disease; CHF, congestive heart failure; CKD, chronic kidney diseases; BMI, body mass index; RBC, red blood cell; Hb, hemoglobin.

**Table 2:** Adjusted ORs for associations between serum 25(OH)D and the risk of anemia.

Serum 25(OH)D	Model 1 OR (95% CI)	Model 2 OR (95% CI)	Model 3 OR (95% CI)
Q1	Ref.	Ref.	Ref.
Q2	0.592 (0.530, 0.661) ^*∗∗∗*^	0.617 (0.552, 0.690) ^*∗∗∗*^	0.735 (0.651, 0.829) ^*∗∗∗*^
Q3	0.396 (0.351, 0.448) ^*∗∗∗*^	0.440(0.388, 0.498) ^*∗∗∗*^	0.527 (0.461, 0.602) ^*∗∗∗*^
Q4	0.463 (0.413, 0.518) ^*∗∗∗*^	0.536 (0.476, 0.603) ^*∗∗∗*^	0.696 (0.611, 0.792) ^*∗∗∗*^
*P* for trend	<0.001	<0.001	<0.001

Q1, 6.31–44.4 nmol/L; Q2, 44.5–60.6 nmol/L; Q3, 60.7–77.7 nmol/L; Q4, 77.8–422 nmol/L; Serum 25(OH)D, serum 25-hydroxyvitamin *D*; ^*∗∗∗*^, *P* < 0.001; OR, odd ratio; CI, confidence interval. Model 1: age and gender. Model 2: model 1 variables plus race/ethnicity, education level, marital status, family poverty income ratio, hypertension, diabetes mellitus, smoke status, and drink status. Model 3 was adjusted for model 2 variables plus the history of coronary heart disease, congestive heart failure, angina pectoris, heart attack, stroke, and chronic kidney diseases, body mass index, waist circumference, folic acid intake, Vitamin B12 intake, Vitamin C intake, iron intake, serum iron, calcium, and phosphorus.

**Table 3:** Subgroup analysis for associations between serum 25(OH)D and the risk of anemia.

Serum 25(OH)D	Q1 OR (95% CI)	Q2 OR (95% CI)	Q3 OR (95% CI)	Q4 OR (95% CI)	*P* for trend	*P* for interaction
Age						
<60	1.00	0.689 (0.589, 0.806) ^*∗∗∗*^	0.470 (0.390, 0.565) ^*∗∗∗*^	0.476 (0.388, 0.583) ^*∗∗∗*^	<0.001	<0.001
≥60	1.00	0.884 (0.726, 1.076)	0.682 (0.557, 0.836) ^*∗∗∗*^	1.073 (0.892, 1.292)	<0.001	

Gender						
Male	1.00	0.759 (0.612, 0.940) ^*∗*^	0.508 (0.404, 0.639) ^*∗∗∗*^	0.866 (0.697, 1.075)	<0.001	<0.001
Female	1.00	0.734 (0.631, 0.853) ^*∗∗∗*^	0.558 (0.471, 0.662) ^*∗∗∗*^	0.620 (0.524, 0.734) ^*∗∗∗*^	<0.001	

Race						
Mexican American	1.00	0.905 (0.663, 1.236)	0.764 (0.527, 1.106)	1.117 (0.724, 1.723)	0.378	<0.001
Other Hispanic	1.00	0.783 (0.488, 1.256)	0.711 (0.428, 1.183)	0.874 (0.499, 1.532)	0.573	
Non-Hispanic Black	1.00	0.989 (0.817, 1.196)	0.742 (0.581, 0.948) ^*∗*^	1.022 (0.802, 1.303)	0.092	
Non-Hispanic White	1.00	1.031 (0.774, 1.373)	0.982 (0.744, 1.296)	1.308 (1.005, 1.703) ^*∗*^	0.019	
Other race	1.00	1.195 (0.783, 1.824)	0.421 (0.243, 0.730) ^*∗∗*^	0.755 (0.463, 1.232)	0.002	

Hypertension						
No	1.00	0.675 (0.567, 0.804) ^*∗∗∗*^	0.471 (0.386, 0.574) ^*∗∗∗*^	0.505 (0.409, 0.625) ^*∗∗∗*^	<0.001	0.001
Yes	1.00	0.791 (0.668, 0.937) ^*∗∗*^	0.579 (0.482, 0.695) ^*∗∗∗*^	0.864 (0.730, 1.022)	<0.001	

DM						
No	1.00	0.680 (0.590, 0.783) ^*∗∗∗*^	0.467 (0.398, 0.547) ^*∗∗∗*^	0.624 (0.535, 0.729) ^*∗∗∗*^	<0.001	<0.001
Yes	1.00	0.921 (0.728, 1.164)	0.728 (0.566, 0.936) ^*∗*^	0.934 (0.733, 1.191)	0.088	

Obesity						
<30 kg/m^2^	1.00	0.761 (0.647, 0.895) ^*∗∗*^	0.538 (0.453, 0.639) ^*∗∗∗*^	0.619 (0.523, 0.732) ^*∗∗∗*^	<0.001	<0.001
≥30 kg/m^2^	1.00	0.718 (0.597, 0.862) ^*∗∗∗*^	0.504 (0.405, 0.627) ^*∗∗∗*^	0.863 (0.701, 1.062)	<0.001	

Q1, 6.31–44.4 nmol/L; Q2, 44.5–60.6 nmol/L; Q3, 60.7–77.7 nmol/L; Q4, 77.8–422 nmol/L; Serum 25(OH)D, serum 25-hydroxyvitamin *D*; ^*∗*^, *P* < 0.05; ^*∗∗*^, *P* < 0.01; ^*∗∗∗*^, *P* < 0.001; OR, odd ratio; CI, confidence interval. Analysis was adjusted for age, gender, race/ethnicity, education level, marital status, family poverty income ratio, hypertension, diabetes mellitus, smoke status, and drink status, the history of coronary heart disease, congestive heart failure, angina pectoris, heart attack, stroke, and chronic kidney diseases, body mass index, waist circumference, folic acid intake, Vitamin B12 intake, Vitamin C intake, iron intake, serum iron, calcium, and phosphorus.

## Data Availability

The survey data are publicly available on the Internet for data users and researchers throughout the world (https://www.cdc.gov/nchs/nhanes/).

## Supplementary Materials

Supplementary Materials Supplementary Figure 1: Restricted cubic spline plot of the association between serum 25(OH)D and the incidence of anemia. Supplementary Figure 2: Associations of serum 25(OH)D with hemoglobin levels and red blood cell counts. (a) Association between serum 25(OH)D and hemoglobin level. (b) Association between serum 25(OH)D and red blood cell count. Supplementary Table 1: Study population data according to serum 25(OH)D quartiles. Supplementary Table 2: Adjusted ORs for associations between serum 25(OH)D and the risk of anemia. Supplementary Table 3: Subgroup analysis for associations between serum 25(OH)D and the risk of anemia.
