# Supplementary material for: The Association between Standardized Serum 25-Hydroxyvitamin D Concentration and Risk of Anemia: A Population-Based Cross-Sectional Study
Source: Int J Clin Pract. 2022 Oct 13;2022:8384306. doi: 10.1155/2022/8384306 (PMC9584730; doi:10.1155/2022/8384306)
Supplement: Supplementary Materials — Supplementary Figure 1: Restricted cubic spline plot of the association between serum 25(OH)D and the incidence of anemia. Supplementary Figure 2: Associations of serum 25(OH)D with hemoglobin levels and red blood cell counts. (a) Association between serum 25(OH)D and hemoglobin level. (b) Association between serum 25(OH)D and red blood cell count. Supplementary Table 1: Study population data according to serum 25(OH)D quartiles. Supplementary Table 2: Adjusted ORs for associations between serum 25(OH)D and the risk of anemia. Supplementary Table 3: Subgroup analysis for associations between serum 25(OH)D and the risk of anemia. [file 8384306.f1.zip › Supplementary Table 1 (2).docx]

| **Supplementary Table 1**. Study population data according to serum 25(OH)D quartiles. | | | | | | |
| --- | --- | --- | --- | --- | --- | --- |
| Serum 25(OH)D | Total | Q1 | Q2 | Q3 | Q4 | *P*-Value |
| Age, years | 62.421 ± 0.659 | 62.395 ± 1.052 | 63.476 ± 1.258 | 62.070 ± 0.976 | 61.944 ± 1.289 | 0.711 |
| Gender, % |  |  |  |  |  | 0.331 |
| Male | 392 (40.9%) | 91 (9.5%) | 103 (10.7%) | 99 (10.3%) | 99 (10.3%) |  |
| Female | 567 (59.1%) | 160 (16.7%) | 133 (13.9%) | 142 (14.8%) | 132 (13.8%) |  |
| Race, % |  |  |  |  |  | < 0.001 |
| Mexican American | 151 (15.7%) | 59 (6.2%) | 47 (4.9%) | 27 (2.8%) | 18 (1.9%) |  |
| Other Hispanic | 18 (1.9%) | 5 (0.5%) | 6 (0.6%) | 4 (0.4%) | 3 (0.3%) |  |
| Non-Hispanic Black | 160 (16.7%) | 87 (9.1%) | 40 (4.2%) | 18 (1.9%) | 15 (1.6%) |  |
| Non-Hispanic White | 600 (62.6%) | 88 (9.2%) | 137 (14.3%) | 188 (19.6%) | 187 (19.5%) |  |
| Other race | 30 (3.1%) | 12 (1.3%) | 6 90.6%） | 4 (0.4%) | 8 (0.8%) |  |
| Family PIR | 2.693 ± 0.098 | 2.425 ± 0.156 | 2.476 ± 0.179 | 2.932 ± 0.154 | 2.813 ± 0.136 | 0.070 |
| Education level, % |  |  |  |  |  | 0.246 |
| Less than high school | 333 (34.7%) | 115 (12.0%) | 89 (9.3%) | 73 (7.6%) | 56 (5.8%) |  |
| High school | 0 (0%) | 0 (0%) | 0 (0%) | 0 (0%) | 0 (0%) |  |
| More than high school | 626 (65.3%) | 136 (14.2%) | 147 (15.3%) | 168 (17.5%) | 175 (18.2%) |  |
| Marital status, % |  |  |  |  |  | 0.234 |
| Having a partner | 537 (56.0%) | 121 (12.6%) | 134 (14.0%) | 144 (15.0%) | 138 (14.4%) |  |
| No partner | 378 (39.4%) | 112 (11.7%) | 92 (9.6%) | 89 (9.3%) | 85 (8.9%) |  |
| Unmarried | 44 (4.6%) | 18 (1.9%) | 10 (1.0%) | 8 (0.8%) | 8 (0.8%) |  |
| Hypertension, % |  |  |  |  |  | 0.088 |
| No | 279 (29.1%) | 49 (5.1%) | 70 (7.3%) | 82 (8.6%) | 78 (8.1%) |  |
| Yes | 680 (70.9%) | 202 (21.1%) | 166 (17.3%) | 159 (16.6%) | 153 (16.0%) |  |
| DM, % |  |  |  |  |  | 0.145 |
| No | 699 (72.9%) | 161(16.8%) | 166 (17.3%) | 192 (20.0%) | 180 (18.8%) |  |
| Yes | 260 (27.1%) | 90 (9.4%) | 70 (7.3%) | 49 (5.1%) | 51 (5.3%) |  |
| Smoker, % |  |  |  |  |  | 0.059 |
| No | 423 (44.1%) | 117 (12.2%) | 99 (10.3%) | 114 (11.9%) | 93 (9.7%) |  |
| Former | 369 (38.5%) | 86 (9.0%) | 86 (9.0%) | 94 (9.8%) | 103 (10.7%) |  |
| Now | 167 (17.4%) | 48 (5.0%) | 51 (5.3%) | 33 (3.4%) | 35 (3.6%) |  |
| Alcohol user, % |  |  |  |  |  | 0.692 |
| Never | 148 (15.4%) | 46 (4.8%) | 29 (3.0%) | 37 (3.9%) | 36 (3.8%) |  |
| Former | 318 (33.2%) | 89 (9.3%) | 89 (9.3%) | 69 (7.2%) | 71 (7.4%) |  |
| Mild | 312 (32.5%) | 61 (6.4%) | 73 (7.6%) | 92 (9.6%) | 86 (9.0%) |  |
| Moderate | 88 (9.2%) | 26 (2.7%) | 20 (2.1%) | 23 (2.4%) | 19 (2.0%) |  |
| Heavy | 93 (9.7%) | 29 (3.0%) | 25 (2.6%) | 20 (2.1%) | 19 (2.0%) |  |
| CHD, % |  |  |  |  |  | 0.041 |
| No | 858 (89.5%) | 218 (22.7%) | 207 (21.6%) | 225 (23.5%) | 208 (21.7%) |  |
| Yes | 101 (10.5%) | 33 (3.4%) | 29 (3.0%) | 16 (1.7%) | 23 (2.4%) |  |
| CHF, % |  |  |  |  |  | 0.135 |
| No | 875 (91.2%) | 223 (23.3%) | 213 (22.2%) | 226 (23.6%) | 213 (22.2%) |  |
| Yes | 84 (8.8%) | 28 (2.9%) | 23 (2.4%) | 15 (1.6%) | 18 (1.9%) |  |
| Angina, % |  |  |  |  |  | < 0.001 |
| No | 864 (90.1%) | 227 (23.7%) | 197 (20.5%) | 226 (23.6%) | 214 (22.3%) |  |
| Yes | 95 (9.9%) | 24 (2.5%) | 39 (4.1%) | 15 (1.6%) | 17 (1.8%) |  |
| Heart attack, % |  |  |  |  |  | 0.236 |
| No | 872 (90.9%) | 225 (23.5%) | 210 (21.9%) | 224 (23.4%) | 213 (22.2%) |  |
| Yes | 87 (9.1%) | 26 (2.7%) | 26 (2.7%) | 224 (23.4%) | 213 (22.2%) |  |
| Stroke, % |  |  |  |  |  | 0.007 |
| No | 885 (92.3%) | 229 (23.9%) | 210 (21.9%) | 227 (23.7%) | 219 (22.8%) |  |
| Yes | 74 (7.7%) | 22 (2.3%) | 26 (2.7%) | 14 (1.5%) | 12 (1.3%) |  |
| CKD, % |  |  |  |  |  | 0.027 |
| No | 625 (65.2%) | 146 (15.2%) | 153 (16.0%) | 170 (17.7%) | 156 (16.3%) |  |
| Yes | 334 (34.8%) | 105 (10.9%) | 83 (8.7%) | 71 (7.4%) | 75 (7.8%) |  |
| Osteoporosis, % |  |  |  |  |  | 0.406 |
| No | 761 (79.4%) | 213 (22.2%) | 192 (20.0%) | 181 (18.9%) | 175 (18.2%) |  |
| Yes | 198 (20.6%) | 38 (4.0%) | 44 (4.6%) | 60 (6.3%) | 56 (5.8%) |  |
| BMI, kg/m^2^ | 30.026 ± 0.289 | 32.650 ± 0.654 | 30.439 ± 0.492 | 29.699 ± 0.536 | 28.143 ± 0.458 | < 0.001 |
| Waist circumference, cm | 103.351 ± 0.619 | 108.486 ± 1.172 | 103.999 ± 0.980 | 102.797 ± 1.235 | 99.709 ± 1.126 | < 0.001 |
| RBC, million cells/ul | 4.576 ± 0.023 | 4.615 ± 0.034 | 4.536 ± 0.034 | 4.604 ± 0.039 | 4.552 ± 0.045 | 0.092 |
| Hb, g/dl | 14.172 ± 0.073 | 14.084 ± 0.095 | 14.086 ± 0.101 | 14.243 ± 0.145 | 14.232 ± 0.120 | 0.589 |
| Serum iron, ug/dl | 82.306 ± 1.183 | 78.179 ± 1.764 | 83.294 ± 3.325 | 82.844 ± 1.843 | 83.900 ± 2.466 | 0.08 |
| Calcium, mg/dl | 9.513 ± 0.019 | 9.501 ± 0.036 | 9.451 ± 0.034 | 9.504 ± 0.028 | 9.582 ± 0.025 | 0.006 |
| Phosphorus, mg/dl | 1.237 ± 0.009 | 1.235 ± 0.017 | 1.231 ± 0.016 | 1.242 ± 0.012 | 1.239 ± 0.013 | 0.966 |
| Mean Energy intake, kcal | 1894.193 ± 31.696 | 1780.319 ± 58.345 | 1866.010 ± 42.089 | 1893.395 ± 50.159 | 2000.016 ± 69.490 | 0.081 |
| Protein intake, g | 73.457 ± 1.449 | 70.254 ± 2.421 | 69.789 ± 2.032 | 75.655 ± 2.917 | 76.458 ± 2.385 | 0.082 |
| Folic acid intake, mcg | 184.707 ± 6.505 | 166.834 ± 10.864 | 192.216 ± 14.420 | 177.736 ± 8.404 | 198.733 ± 14.743 | 0.439 |
| Vitamin B12 intake, mcg | 5.353 ± 0.281 | 4.469 ± 0.383 | 5.221 ± 0.378 | 5.148 ± 0.291 | 6.313 ± 0.611 | 0.049 |
| Vitamin C intake, mg | 82.785 ± 3.003 | 82.029 ± 5.790 | 74.910 ± 6.016 | 90.080 ± 4.374 | 82.119 ± 4.174 | 0.078 |
| Iron intake, mg | 14.982 ± 0.364 | 13.741 ± 0.458 | 14.674 ± 0.615 | 15.342 ± 0.519 | 15.749 ± 0.773 | 0.026 |
| PTH, pg/ml | 49.522 ± 1.685 | 61.508 ± 2.887 | 54.397 ± 3.828 | 47.250 ± 1.960 | 39.295 ± 1.453 | < 0.001 |
| Arthritis or rheumatism | 4747.182 ± 155.410 | 4606.596 ± 370.283 | 4682.901 ± 344.188 | 4700.719 ± 254.208 | 4949.712 ± 254.757 | 0.889 |
| problem, days |  |  |  |  |  |  |
| Anemia, % |  |  |  |  |  | 0.136 |
| No | 853 (88.9%) | 213 (22.2%) | 207 (21.6%) | 223 (23.3%) | 210 (21.9%) |  |
| Yes | 106 (11.1%) | 38 (4.0%) | 29 (3.0%) | 18 (1.9%) | 21 (2.2%) |  |

Abbreviations: Q1, 9.1–42.2 nmol/L; Q2, 42.3–55.7 nmol/L; Q3, 55.8–70.4 nmol/L; Q4, 70.5–137 nmol/L; Serum 25(OH)D, serum 25-hydroxyvitamin D; Family PIR, family poverty income ratio; DM, diabetes mellitus; CHD, coronary heart disease; CHF, congestive heart failure; CKD, chronic kidney diseases; BMI, body mass index; RBC, red blood cell; Hb, hemoglobin; PTH, [parathyroid hormone](javascript:;).
