# Supplementary material for: The Association between Standardized Serum 25-Hydroxyvitamin D Concentration and Risk of Anemia: A Population-Based Cross-Sectional Study
Source: Int J Clin Pract. 2022 Oct 13;2022:8384306. doi: 10.1155/2022/8384306 (PMC9584730; doi:10.1155/2022/8384306)
Supplement: Supplementary Materials — Supplementary Figure 1: Restricted cubic spline plot of the association between serum 25(OH)D and the incidence of anemia. Supplementary Figure 2: Associations of serum 25(OH)D with hemoglobin levels and red blood cell counts. (a) Association between serum 25(OH)D and hemoglobin level. (b) Association between serum 25(OH)D and red blood cell count. Supplementary Table 1: Study population data according to serum 25(OH)D quartiles. Supplementary Table 2: Adjusted ORs for associations between serum 25(OH)D and the risk of anemia. Supplementary Table 3: Subgroup analysis for associations between serum 25(OH)D and the risk of anemia. [file 8384306.f1.zip › Supplementary Table 3 (2).docx]

| **Supplementary Table 3.** Subgroup analysis for associations between serum 25(OH)D and the risk of anemia. | | | | | | |
| --- | --- | --- | --- | --- | --- | --- |
| Serum 25(OH)D | Q1 | Q2 | Q3 | Q4 | *P* for trend | *P* for interaction |
|  | OR (95%CI) | OR (95%CI) | OR (95%CI) | OR (95%CI) |  |  |
| Age |  |  |  |  |  | 0.399 |
| < 60 | 1.00 | 1.003 (0.226, 4.458) | 0.282 (0.046, 1.731) | 0.332 (0.051, 2.173) | 0.396 |  |
| ≥ 60 | 1.00 | 0.742 (0.413, 1.331) | 0.505 (0.256, 0.996) * | 0.640 (0.329, 1.248) | 0.247 |  |
| Gender |  |  |  |  |  | 0.009 |
| Male | 1.00 | 0.962 (0.439, 2.108) | 0.431 (0.167, 1.114) | 0.762 (0.319, 1.819) | 0.317 |  |
| Female | 1.00 | 0.641 (0.293, 1.404) | 0.511 (0.216, 1.211) | 0.389 (0.152, 0.998) * | 0.209 |  |
| Race |  |  |  |  |  | 0.064 |
| Mexican American | 1.00 | - | - | - | - |  |
| Other Hispanic | 1.00 | - | - | - | - |  |
| Non-Hispanic Black | 1.00 | 0.681 (0.245, 1.897) | 0.591 (0.138, 2.524) | 1.449 (0.376, 5.578) | 0.648 |  |
| Non-Hispanic White | 1.00 | 1.145 (0.444, 2.958) | 0.651 (0.245, 1.733) | 0.807 (0.313, 2.085) | 0.580 |  |
| Other race | 1.00 | - | - | - | - |  |
| Hypertension |  |  |  |  |  | 0.096 |
| No | 1.00 | 3.423 (0.642, 18.238) | 1.841 (0.329, 10.310) | 2.786 (0.511, 15.181) | 0.456 |  |
| Yes | 1.00 | 0.608 (0.329, 1.122) | 0.382 (0.183, 0.797) * | 0.409 (0.195, 0.858) * | 0.028 |  |
| DM |  |  |  |  |  | 0.568 |
| No | 1.00 | 0.782 (0.374, 1.632) | 0.480 (0.217, 1.062) | 0.513 (0.233, 1.131) | 0.221 |  |
| Yes | 1.00 | 0.680 (0.284, 1.628) | 0.476 (0.150, 1.505) | 0.715 (0.236, 2.164) | 0.609 |  |
| Obesity |  |  |  |  |  | 0.128 |
| < 30 kg/m^2^ | 1.00 | 0.835 (0.387, 1.802) | 0.654 (0.287, 1.489) | 0.738 (0.334, 1.630) | 0.775 |  |
| ≥ 30 kg/m^2^ | 1.00 | 0.693 (0.310, 1.550) | 0.353 (0.119, 1.047) | 0.452 (0.145, 1.409) | 0.230 |  |

Abbreviations: Q1, 9.1–42.2 nmol/L; Q2, 42.3–55.7 nmol/L; Q3, 55.8–70.4 nmol/L; Q4, 70.5–137 nmol/L; Serum 25(OH)D, serum 25-hydroxyvitamin D; **P* < 0.05; OR, odd ratio; CI, confidence interval. Model 1: age and gender. Model 2: model 1 variables plus race/ethnicity, education level, marital status, family poverty income ratio, hypertension, diabetes mellitus, smoke status, and drink status. Model 3 was adjusted for model 2 variables plus the history of coronary heart disease, congestive heart failure, angina pectoris, heart attack, stroke, chronic kidney diseases and osteoporosis, body mass index, waist circumference, folic acid intake, Vitamin B12 intake, Vitamin C intake, iron intake, serum iron, calcium, phosphorus, PTH, and arthritis or rheumatism problem.
