# Supplementary figures and images for: The Association between Standardized Serum 25-Hydroxyvitamin D Concentration and Risk of Anemia: A Population-Based Cross-Sectional Study
Source: Int J Clin Pract. 2022 Oct 13;2022:8384306. doi: 10.1155/2022/8384306 (PMC9584730; doi:10.1155/2022/8384306)

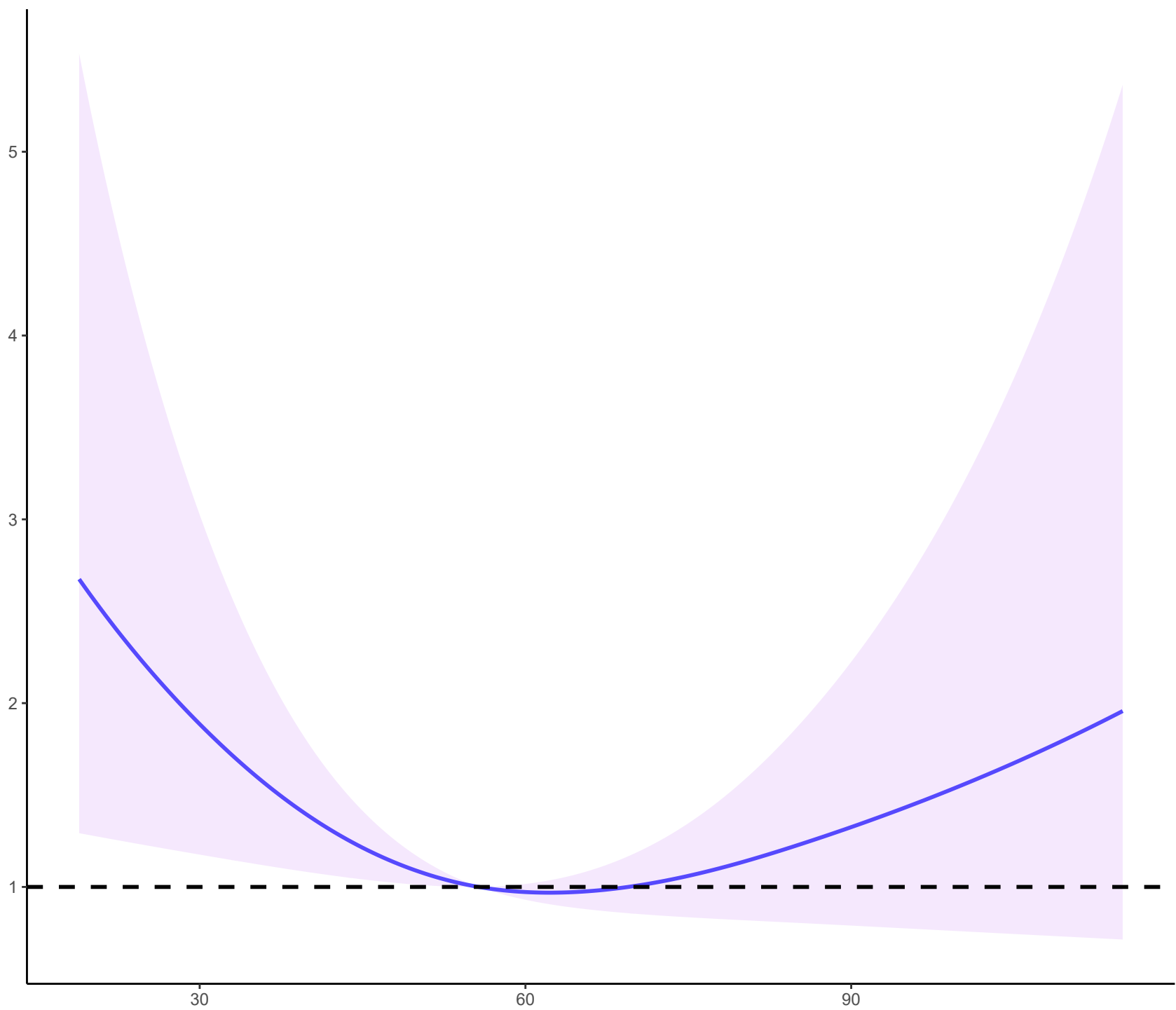

Supplement: Supplementary Materials — Supplementary Figure 1: Restricted cubic spline plot of the association between serum 25(OH)D and the incidence of anemia. Supplementary Figure 2: Associations of serum 25(OH)D with hemoglobin levels and red blood cell counts. (a) Association between serum 25(OH)D and hemoglobin level. (b) Association between serum 25(OH)D and red blood cell count. Supplementary Table 1: Study population data according to serum 25(OH)D quartiles. Supplementary Table 2: Adjusted ORs for associations between serum 25(OH)D and the risk of anemia. Supplementary Table 3: Subgroup analysis for associations between serum 25(OH)D and the risk of anemia. [file 8384306.f1.zip › Supplementary Figure 1 (1).pdf]

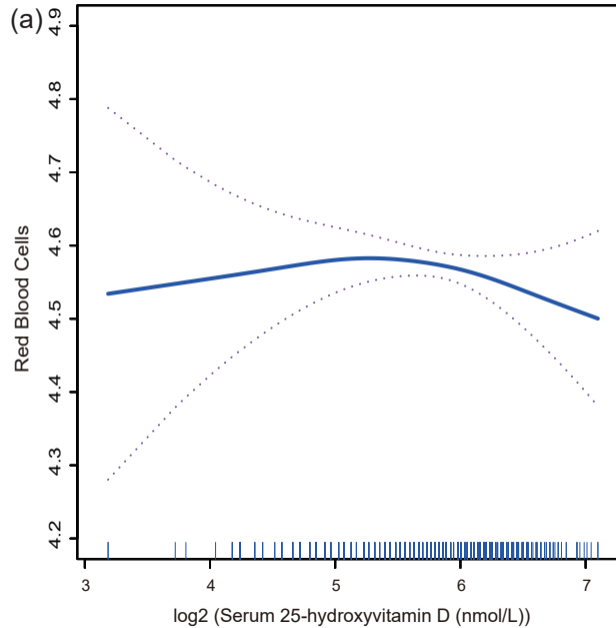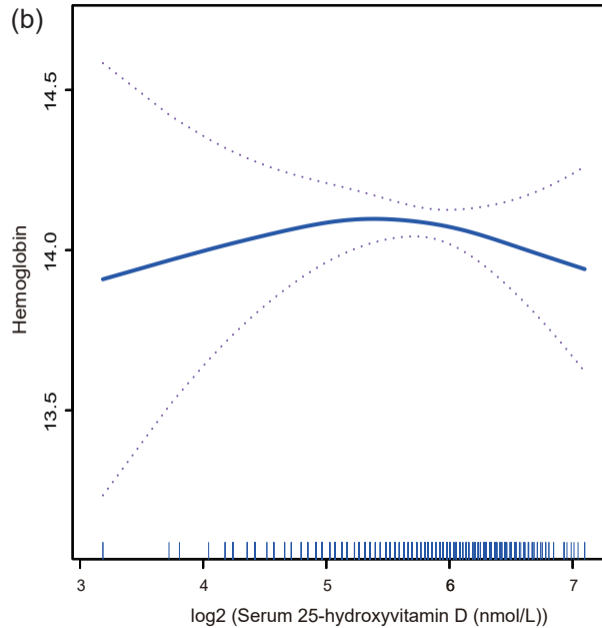

Supplement: Supplementary Materials — Supplementary Figure 1: Restricted cubic spline plot of the association between serum 25(OH)D and the incidence of anemia. Supplementary Figure 2: Associations of serum 25(OH)D with hemoglobin levels and red blood cell counts. (a) Association between serum 25(OH)D and hemoglobin level. (b) Association between serum 25(OH)D and red blood cell count. Supplementary Table 1: Study population data according to serum 25(OH)D quartiles. Supplementary Table 2: Adjusted ORs for associations between serum 25(OH)D and the risk of anemia. Supplementary Table 3: Subgroup analysis for associations between serum 25(OH)D and the risk of anemia. [file 8384306.f1.zip › Supplementary Figure 2 (1).pdf]
